# Digital Phenotyping for Monitoring and Disease Trajectory Prediction of Patients With Cancer: Protocol for a Prospective Observational Cohort Study

**DOI:** 10.2196/49096

**Published:** 2023-10-10

**Authors:** Gabrielė Jenciūtė, Gabrielė Kasputytė, Inesa Bunevičienė, Erika Korobeinikova, Domas Vaitiekus, Arturas Inčiūra, Laimonas Jaruševičius, Romas Bunevičius, Ričardas Krikštolaitis, Tomas Krilavičius, Elona Juozaitytė, Adomas Bunevičius

**Affiliations:** 1 Faculty of Informatics, Vytautas Magnus University Kaunas Lithuania; 2 Faculty of Political Science and Diplomacy, Vytautas Magnus University Kaunas Lithuania; 3 Oncology Institute, Lithuanian University of Health Sciences Kaunas Lithuania; 4 ProIT Vilnius Lithuania; 5 Department of Neurology, Columbia University Vagelos College of Physicians and Surgeons New York, NY United States

**Keywords:** cancer, digital phenotyping, biomarkers, oncology, digital phenotype, biomarker, data collection, data generation, monitor, monitoring, data collection, predict, prediction, model, models, mobile phone

## Abstract

**Background:**

Timely recognition of cancer progression and treatment complications is important for treatment guidance. Digital phenotyping is a promising method for precise and remote monitoring of patients in their natural environments by using passively generated data from sensors of personal wearable devices. Further studies are needed to better understand the potential clinical benefits of digital phenotyping approaches to optimize care of patients with cancer.

**Objective:**

We aim to evaluate whether passively generated data from smartphone sensors are feasible for remote monitoring of patients with cancer to predict their disease trajectories and patient-centered health outcomes.

**Methods:**

We will recruit 200 patients undergoing treatment for cancer. Patients will be followed up for 6 months. Passively generated data by sensors of personal smartphone devices (eg, accelerometer, gyroscope, GPS) will be continuously collected using the developed LAIMA smartphone app during follow-up. We will evaluate (1) mobility data by using an accelerometer (mean time of active period, mean time of exertional physical activity, distance covered per day, duration of inactive period), GPS (places of interest visited daily, hospital visits), and gyroscope sensors and (2) sociability indices (frequency of duration of phone calls, frequency and length of text messages, and internet browsing time). Every 2 weeks, patients will be asked to complete questionnaires pertaining to quality of life (European Organization for Research and Treatment of Cancer Core Quality of Life Questionnaire [EORTC QLQ-C30]), depression symptoms (Patient Health Questionnaire-9 [PHQ-9]), and anxiety symptoms (General Anxiety Disorder-7 [GAD-7]) that will be deployed via the LAIMA app. Clinic visits will take place at 1-3 months and 3-6 months of the study. Patients will be evaluated for disease progression, cancer and treatment complications, and functional status (Eastern Cooperative Oncology Group) by the study oncologist and will complete the questionnaire for evaluating quality of life (EORTC QLQ-C30), depression symptoms (PHQ-9), and anxiety symptoms (GAD-7). We will examine the associations among digital, clinical, and patient-reported health outcomes to develop prediction models with clinically meaningful outcomes.

**Results:**

As of July 2023, we have reached the planned recruitment target, and patients are undergoing follow-up. Data collection is expected to be completed by September 2023. The final results should be available within 6 months after study completion.

**Conclusions:**

This study will provide in-depth insight into temporally and spatially precise trajectories of patients with cancer that will provide a novel digital health approach and will inform the design of future interventional clinical trials in oncology. Our findings will allow a better understanding of the potential clinical value of passively generated smartphone sensor data (digital phenotyping) for continuous and real-time monitoring of patients with cancer for treatment side effects, cancer complications, functional status, and patient-reported outcomes as well as prediction of disease progression or trajectories.

**International Registered Report Identifier (IRRID):**

PRR1-10.2196/49096

## Introduction

Cancer continues to be the leading cause of morbidity and mortality worldwide and a serious public health challenge [1-3]. Early cancer detection as well as increased effectiveness and availability of novel treatment approaches such as immunotherapy and targeted therapies have resulted in a steadily growing number of cancer survivors [4-7]. Continuous monitoring of patients undergoing treatment for cancer and cancer survivors is important for timely identification of cancer treatment complications and cancer progression that is important for optimized long-term prognosis [8]. Therefore, there is an urgent need for developing widely available, automated, and evidence-based monitoring systems of patients with cancer [9].

The COVID-19 pandemic continues to be a serious public health challenge [10]. Patients with cancer are vulnerable to SARS-CoV-2 infection due to immunosuppression, frequent hospital visits [11,12], and lower efficacy of the COVID-19 vaccines [13]. The mortality risk of patients with cancer infected with SARS-CoV-2 was greater than that of the general population and was shown to exceed by 33% among patients with thoracic and hematologic malignancies [14-16]. A meta-analysis of 52 studies and 18,650 patients estimated the probability of death at 25.6% in patients with cancer infected with COVID-19 [17]. Furthermore, the COVID-19 pandemic caused major delays and interruptions in cancer treatments [18,19], and they are estimated to have caused over 15% increase in additional cancer deaths [20]. Finally, patients with cancer were vulnerable to adverse mental health and quality of life sequalae during the COVID-19 pandemic [21,22].

Although there is no standardized definition for mobile health (mHealth), the World Health Organization defines mHealth as “medical or public health practice that is delivered with the support of mobile phones, patient monitoring devices, and other wireless devices.” mHealth encompasses the use of mobile phones and other wireless technologies for health care and is a promising approach for improving health monitoring and delivery of health interventions [23-25]. The adoption of telehealth and mHealth solutions is growing [26]. The number of digital health apps currently exceeds 300,000, with hundreds of apps being introduced every day [27]. The market size of digital health apps will grow exponentially in the coming decade [27]. However, the adoption of digital health apps in clinical practice is limited due to numerous obstacles such as unclear clinical value because they are often not developed by health care professionals and not tested in clinical environments [28]. Thus, there is an urgent need to develop evidence-based personalized telehealth or mHealth solutions and test them in real-word clinical environments that would allow evidence-based monitoring of vulnerable patients with cancer and would ideally consider the clinical, functional, and patient-reported health outcomes [29-31].

Digital phenotyping allows continuous and spatially and temporally precise quantification of individual’s phenotype in his or her natural environment by using passively harnessed data from personal digital devices, including smartphone sensors [32,33]. Digital phenotyping approaches have been increasingly tested across mental health and somatic conditions [34], including amyotrophic lateral sclerosis [35], multiple sclerosis [36], posttraumatic stress disorder [37], depression [38], schizophrenia [39], sleep disorders [40], and spine disorders [41]. There is a growing body of evidence that passively generated data can allow identification of treatment complications and disease trajectories of patients with cancer. For example, the Helping Our Patients Excel study of 10 patients (mean age 60 [SD 11] years and mean Eastern Cooperative Oncology Group performance status of 1 [SD 0.66]) with gynecological cancer undergoing palliative chemotherapy found that passively acquired accelerometer data using wearable devices enabled the identification of high-risk patients and complications [42]. In another prospective observational study, 62 individuals undergoing elective cancer surgery were followed up using a smartphone app for a median of 147 (IQR 77-179) days after the surgery [43]. They found that exertional physical activity in patients who experienced postoperative events (27%) was lower than that in those who did not experience postoperative events, suggesting that this approach can allow to quantify recovery after cancer surgery [43]. However, these studies are limited by small to moderate sample sizes and consideration of a snapshot of cancer journey. Numerous barriers remain for implementing mobile-sensing data into the care of patients with cancer due to ongoing challenges related to raw data analysis and interpretation as well as integration of results in the clinical workflow [44]. Interpretation of passively collected data within clinical contexts requires multidisciplinary collaboration to identify potential clinical values of the mobile-sensing data. The analysis of passively harnessed sensing data is another important challenge, and it has been shown that deep learning approaches can be used to anticipate complications of patients undergoing treatment for hematologic malignancies [45]. Therefore, future studies are warranted to better understand the potential clinical values of mobile sensing data in oncology.

The aim of our study is to evaluate the usefulness of passively generated smartphone sensor data streams (as in digital phenotyping) for monitoring patients with cancer. We will evaluate if novel cancer trajectory biomarkers that integrate high-resolution passively acquired data streams from smartphone sensors with actively collected data can enable real-time data analytics and monitoring of patients with cancer in their natural environment. We will (1) develop and adapt a monitoring system tailored for patients with cancer that integrates passively and actively acquired data streams, (2) investigate if continuous monitoring of patients with cancer using passively collected data acquired through personal smartphones correlates with objective and patient-reported health status and therefore can be used as actionable digital phenotype, and (3) develop prediction models anticipating disease trajectories as well as functional and reported outcomes of patients with cancer. This study will allow to better understand the potential clinical value of passively generated smartphone sensor data (digital phenotyping) for continuous and real-time monitoring of patients with cancer for treatment side effects, cancer complications, functional status, and patient-reported outcomes, as well as prediction of disease progression or trajectories.

## Methods

### Study Design

This is a prospective observational cohort study with a 6-month follow-up of patients diagnosed with cancer who will use a smartphone app to acquire continuously generated data from smartphone sensors (passively generated data) and deploy questionnaires (actively generated data). This study is conducted at the Department of Oncology, Hospital of Lithuanian University of Health Sciences, Kaunas, Lithuania, in collaboration with Vytautas Magnus University, Kaunas, Lithuania, and ProIT, Vilnius, Lithuania.

### Patient and Public Involvement

Patient involvement in this study will last for the duration of the study and will include continuous collection of smartphone sensor data, actively collected questionnaire data via LAIMA app, and 3 in-person visits. The patients and the public were not involved in the design of this study.

### Recruitment

The study inclusion criteria are (1) diagnosis of cancer, (2) current active treatment (radiation therapy, chemotherapy) for cancer or surveillance, (3) ability to comprehend Lithuanian, (4) ownership of a smartphone device (Android or iOS platform) that can support the smartphone apps, (5) age of 18 years or older, and (6) ability to provide informed consent. Patients will be excluded if they have cognitive, visual, or functional impairment or any other clinically significant medical condition that in the opinion of the study investigators could affect participant safety, preclude evaluation of responses, or interfere with the ability to comply with the study procedures.

### Ethics Approval

Ethics approval was obtained from the Kaunas Regional Bioethics Committee on April 14, 2022 (protocol BE-2-31). All participants will provide informed consent before inclusion in this study. Identifying data will be available for the study oncologists and will be deidentified for other members of the research team. Patients will not receive compensation for participating in this study.

### Sample Size

The sample size was calculated using the Digital Phenotyping Power Calculator tool [46]. We plan to recruit 200 patients diagnosed with cancer undergoing active treatment and actively follow-up with them for at least 3 months, with expected missing data of <50% that will allow to achieve power (type 1 error .05) of .971 at effect size (β₁) of .03 and power of 1 and effect sizes (β₁) of .05 and .07.

### Primary Outcome

We will examine changes in each of the collected digital biomarker data streams during the 6-month follow-up period in relation to progression-free survival. Cancer progression will be defined based on clinical, laboratory, and radiological data in accordance with guidelines and criteria for the specific cancer type. Cancer progression will be determined by the treating oncologist according to relevant clinical guidelines.

### Secondary Outcome

We will examine temporal changes in questionnaire data trajectories collected using the LAIMA app and will examine the association of passively harnessed digital biomarkers with actively collected questionnaire data. We will also examine the association of passively acquired digital biomarker data with clinically relevant outcomes of patients with cancer, such as overall survival, cancer-related complications (type of complication, unexpected presentation to emergency department or hospital admission), and cancer treatment–related adverse events. The precision of digital biomarker data to identify and predict clinically relevant outcomes of patients with cancer will be examined.

### Digital Data

Data will be collected using the LAIMA app that was developed by the researchers based on the open-source Beiwe platform [47]. The LAIMA app is supported by iOS and Android operating systems. We also developed a corresponding digital platform that is linked with the smartphone app and will be used for patient registration and remote monitoring by the study investigators.

The LAIMA app and digital platform will be used to collect and integrate (1) passively generated data streams from smartphone sensors that will be uploaded to the LAIMA platform and integrated with questionnaires and clinical data, (2) actively collected questionnaire data that study participants will be prompted to answer in predefined days at 2-week intervals after inclusion in this study, and (3) sociodemographic and clinical data that will be entered by the study investigators at initial and follow-up study visits. In this study, we will include questionnaires that are commonly used for clinical and cancer research and were validated in Lithuanian as well as clinical data that are relevant for patients with cancer. The data will be uploaded to the server and aggregated. We utilize cloud services for secure data storage as well as data preparation and predictive modeling using generated data. The LAIMA platform has several data preparation methods such as classification, clustering, regression, and ranking. The platform also has a built-in artificial intelligence algorithm function. The LAIMA app and platform are compliant with the European Union General Data Protection Regulation requirements [48]. Continuously and passively generated data from smartphone sensors will be collected during the course of this study that will take from 3 to 6 months for each patient included in this study. The following smartphone sensor data will be considered for this study:

1. Mobility data will include continuous collection and aggregation of passively generated data streams from the accelerometer (mean time of active period, mean time of exertional physical activity, distance covered per day, duration of inactive period), GPS (places of interest visited daily, hospital visits), and gyroscope.
2. Sociability indices will include continuous capture and aggregation of frequency of duration of phone calls, frequency and length of text messages, and internet browsing time.

### Questionnaires

#### Eastern Cooperative Oncology Group Questionnaire

The Eastern Cooperative Oncology Group questionnaire will be used for the assessment of the functional status of patients with cancer during their clinic visits [49]. It is a widely used measure of the functional status of patients with cancer in clinical practice and in clinical trials.

#### European Organization for Research and Treatment of Cancer Core Quality of Life Questionnaire

The European Organization for Research and Treatment of Cancer Core Quality of Life Questionnaire (EORTC QLQ-C30) will be used for the assessment of quality of life of the study participants [50,51]. The EORTC QLQ-C30 will be deployed during study visits and via the LAIMA app. The EORTC QLQ-C30 consists of 9 multi-item functional scales and 6 single-item symptom scales that evaluate global health status, functional status, role functioning, emotional functioning, cognitive functioning, social functioning, fatigue, nausea and vomiting, pain, dyspnea, insomnia, appetite loss, constipation, diarrhea, and financial difficulties. Scores are linearly transformed to a scale ranging from 0 to 100, with higher scores indicating better health-related quality of life on global health status and functional status scales and worse health-related quality of life on symptom scales.

#### Patient Health Questionnaire-9

The Patient Health Questionnaire-9 (PHQ-9) will be used for the assessment of depressive symptom severity during study visits and via the LAIMA app [52,53]. The PHQ-9 consists of 9 symptoms of depression that respondents rate based on their severity during the preceding 2 weeks, with a greater score indicating greater depressive symptom severity and with scores of 10 or greater indicating moderate to severe depressive symptoms.

#### General Anxiety Disorder-7 Questionnaire

The General Anxiety Disorder-7 (GAD-7) scale will be used for the assessment of anxiety symptom severity during the study visit and via the LAIMA app [54,55]. The GAD-7 includes 7 questions pertaining to generalized anxiety disorder, with higher scores indicating greater anxiety symptom severity.

#### COVID-19 Fears Questionnaire

The COVID-19 Fears Questionnaire for chronic medical conditions will be used for evaluating fears related to the COVID-19 pandemic [56,57]. This questionnaire includes 10 items. Respondents are asked to select the response that reflects how much each statement describes their experience on a typical day in the last week on a 5-point numerical scale ranging from 1 (not at all) to 5 (extremely). The total score is the sum of all items and ranges from 10 to 50, with higher scores reflecting greater COVID-19 fear. The questionnaire has been validated in Lithuania [56] and will be administered during the study visits.

#### Questionnaire Administration

The Eastern Cooperative Oncology Group questionnaire, EORTC QLQ-C30, PHQ-9, GAD-7, and the COVID-19 Fears Questionnaire for chronic medical conditions will be administered by trained clinicians. Deidentified data will be entered into a database, and hard copy documents are kept on-site under lock and key. EORTC QLQ-C30, PHQ-9, GAD-7 will also be deployed via the LAIMA app to study participants in predefined 2-week intervals via text messages and a secure link to the website. Participants will have 2 days to complete each questionnaire. The data will be uploaded to the LAIMA digital platform and integrated with other data sources for each patient.

### Sociodemographic Data

We will consider patients’ age at the time of cancer diagnosis and study inclusion, gender, education, job, and marital status. The type of smartphone device and operating system (Android, iOS or other) will be recorded.

### Clinical Data

Information about cancer diagnosis, cancer location, presence of distant metastases, and tumor, node, metastasis stage at time of study inclusion, date of cancer diagnosis, and pathology results, including relevant molecular data, will be recorded at the initial study visit. We will record information regarding currently active cancer treatment(s), including radiation therapy (dose, target, and number of fractions) and chemotherapy (chemotherapeutic agent and dose and treatment start and finish dates). We will also gather information about all previous cancer treatments (surgery, radiation therapy, and chemotherapy), including their date, radiation dose, and chemotherapeutic agent(s). Information about comorbidities and currently used medication will be considered.

At 2 follow-up visits that will take place at 1-3 months and 3-6 months after patient inclusion in the study, we will record information pertaining to cancer treatment (chemotherapy, radiation therapy, and other) that is ongoing or that the patient has received during the follow-up period. Disease progression and its date will be evaluated using standard clinical assessment studies and relevant criteria. Treatment complications (date and description) and unanticipated visits to the emergency department (date and description) will be recorded for all study patients. Disease progression and complications of patients who will not be able to attend the follow-up visit will be evaluated by reviewing medical records or via phone interviews. All clinical and sociodemographic data will be recorded and entered into the LAIMA platform by the study investigators who are board-certified oncologists.

### Detailed Procedure

#### Baseline Visit

At the baseline visit, the study investigator will inform the patients about the study and procedures, and informed consent will be obtained. Participants will complete a battery of questionnaires that will be administered by the study investigators (Table 1). The LAIMA app will be installed into the participant’s personal smartphone device, and an account will be created. Participants will be provided with a personal password and informed about the procedures in case he or she decides to withdraw from the study. The study investigators will ensure that the passively acquired data are being properly collected from a smartphone device by registering in the LAIMA platform.

**Table 1:** Study protocol.

		Visit 1 (month 0)	Data collected using smartphone app	Visit 2 (months 1-3)	Visit 3 (months 3-6)
**Data collected only at initial visit**					
	Informed consent	✓			
	Installation of mobile app	✓			
	Demographic and sociodemographic data	✓			
	Clinical data	✓		✓	✓
	Treatment complications and adverse events	✓		✓	✓
	Functional status (Eastern Cooperative Oncology Group)	✓		✓	✓
	Health-related quality of life (EORTC QLQ-C30^a^)	✓		✓	✓
	Symptoms of depression (Patient Health Questionnaire-9)	✓		✓	✓
	Symptoms of anxiety (General Anxiety Disorder-7)	✓		✓	✓
	COVID-19 Fear (COVID-19 Fear Questionnaire)	✓		✓	✓
**Questionnaire data collected using smartphone app (every 2 weeks)**					
	Health-related quality of life (EORTC QLQ-C30)		✓		
	Symptoms of depression (Patient Health Questionnaire-9)		✓		
	Symptoms of anxiety (General Anxiety Disorder-7)		✓		
**Passively collected data using smartphone app (continuous)**					
	Accelerometer		✓		
	Gyroscope		✓		
	GPS		✓		
	Phone on or off		✓		
	Phone calls		✓		
	Phone calls		✓		
	Text messages		✓		
	Internet browsing time		✓		

^a^EORTC QLQ-C30: European Organization for Research and Treatment of Cancer Core Quality of Life questionnaire.

#### Follow-up Visits

All study patients will complete follow-up visits at months 1 to 3 and months 3 to 6 after the study inclusion. Follow-up visits will take place during routine clinical visits or via telephone encounter. We anticipate that most follow-up encounters will take place during in-person clinic visits. At follow-up visits, patients will complete the study questionnaires, and relevant clinical, laboratory, and imaging data will be reviewed for assessment of disease progression and treatment-related adverse events (Table 1). Given the observational nature of this study, patients will undergo clinical, laboratory, and imaging evaluation as clinically indicated and per appropriate guidelines because we will include patients with different cancer diagnoses undergoing different treatments. At the end of month 6 of the study or in case of patient death, the app will be remotely uninstalled from the device. Monitoring of passively generated data via smartphone sensors and actively collected questionnaire data via LAIMA app collection and upload in the platform will be conducted daily. Patients without passively generated or uploaded data for 3 days or more will be contacted by the study investigators, and problems will be resolved.

### Data Analysis

Data cleaning will be performed on both active and passive data. The main cleaning tasks will be removing errors, identifying outliers, and filling the missing data. Moreover, data will be transformed using aggregation and normalization methods. Missing data from smartphone sensors will be restored using data recovery extension (Firebase Cloud Messaging). For small numbers of missing observations, the missing values will be inputted using linear or polynomial interpolation methods (data imputation will not be used for mixed effects regression models). Cases with more than half of the smartphone sensor data missing will be excluded from the analyses. Missing answers in a questionnaire will not be considered in the analyses, and data imputation will not be performed as it could skew the results of the analyses. A descriptive analysis (mean, SD, median, relative frequencies for qualitative variables) of all the variables will be performed. To investigate associations among digital, clinical, and patient-reported health outcomes, *χ*^2^ test (qualitative characteristics) or 2-sided *t* test or Mann-Whitney (quantitative characteristics) *U* test will be conducted. To visualize data, boxplots, bar plots, and heatmaps will be used. In the initial exploratory analyses, we will analyze each digital biomarker separately that will be followed by their aggregation to identify the most informative biomarkers and their sets for different clinical and patient-reported outcome measures of patients with cancer. Mixed effects regression models will be used for the analysis of longitudinal data obtained from smartphone sensors. For all hypotheses, statistically significant differences will be evaluated at a significance level of .05, and multiple comparison adjustments will be performed. Statistical analysis will be employed using SPSS software (version 27; IBM Corp) and RStudio (version 2022.12.0+353; Posit, PBC).

## Results

Patient recruitment and data collection are expected to be completed by September 2023. As of July 2023, we have reached the planned recruitment target, and patients are undergoing follow-up. The final results of this study should be available within 6 months after the study completion.

## Discussion

In this prospective observational cohort study, 200 patients with cancer will be followed up for 6 months by using (1) continuously and passively generated data from sensors (accelerometer, gyroscope, GPS) of smartphone devices (digital phenotyping) and (2) actively collected data using questionnaires for assessment of quality of life, symptoms, depression, anxiety, and COVID-19 fear. Disease progression and treatment complications will be evaluated during clinic visits. This study will allow to better understand the potential value of passively generated data from smartphone sensors for remote real-time monitoring of patients with cancer and anticipation of disease progression or trajectories, treatment complications, and unfavorable patient-reported outcomes.

Digital phenotyping is a promising approach in oncology that enables utilization of data that are passively generated by patients in their natural environments. It is a user-friendly approach because it uses personal digital devices, thus causing limited interruption of daily routine and device use habits. High and growing adoption of smartphones across age groups underscores the scalability of digital phenotyping approaches [58]. However, the barriers for deploying and integrating the digital phenotyping approach in oncology workflow should be considered and addressed, including data privacy and security concerns and participant willingness to share data. Caveats of analysis of passively generated data and its integration with routinely collected clinical and patient-reported data should be overcome to provide clinically meaningful digital biomarkers that can provide additional clinical value above and beyond the standard of care of patients with cancer. Emerging evidence suggests that this approach can be promising to predict the complications of cancer treatment [42,43]. However, it remains largely unclear if this approach can accurately prognosticate disease and treatment trajectories of patients with cancer. Our findings will allow us to better understand the potential clinical value of digital biomarkers for the prediction of clinical and patient-centered trajectories of patients with cancer.

The limitation of this study is the inclusion of patients with different cancer diagnoses and undergoing various treatments that can have a direct impact on the digital biomarkers included in this study. However, this will allow us to better understand which patients could potentially benefit the most from such a monitoring approach. The use of sensors of personal smartphone devices will provide real-world data with regard to patient behavior in their natural home environment but is subjected to individual differences of smartphone use habits. We hope that our study will provide further insights regarding the value of digital biomarkers and will be used to inform the planning and implementation of interventional clinical trials in oncology.

## Abbreviations

EORTC QLQ-C30: European Organization for Research and Treatment of Cancer Core Quality of Life Questionnaire
GAD-7: General Anxiety Disorder-7
mHealth: mobile health
PHQ-9: Patient Health Questionnaire-9

## Data Availability

Data will be available 6 months after study completion.
